# Extracellular Proteins of *Mycoplasma synoviae*

**DOI:** 10.5402/2012/802308

**Published:** 2012-09-06

**Authors:** Manuel Sebastián Rebollo Couto, Catia Silene Klein, Daiane Voss-Rech, Hernán Terenzi

**Affiliations:** ^1^Centro de Biologia Molecular Estrutural, Departamento de Bioquímica, CCB, Universidade Federal de Santa Catarina, 88040-900 Florianópolis, SC, Brazil; ^2^Embrapa Swine and Poultry Research Center, Animal Health Laboratory, 89700-000 Concordia, SC, Brazil

## Abstract

*Mycoplasma synoviae* is a Gram positive bacteria lacking of cell wall that affects chickens and turkeys causing infection in the upper respiratory tract and in some cases arthritis, with economical impact to broiler breeders. Treatment and prevention of avian synovitis depend on knowledge of the infectious process. Secreted or surface-exposed proteins play a critical role in disease because they often mediate interactions between host and pathogen. In the present work, we sought to identify possible *M. synoviae* secreted proteins by cultivating the bacteria in a modified protein-free Frey medium. Using this approach, we were able to detect in the cell-free fraction a number of proteins that have been shown in other organisms to be secreted, suggesting that they may also be secreted by *M. synoviae*.

## 1. Introduction

The growth of poultry industry is often limited by infectious diseases that affect birds. *Mycoplasma synoviae* is a major avian extracellular pathogen associated with synovitis in chickens and turkeys [1, 2]. Disease can occur as chronical subclinical to severe upper respiratory infection and, under unknown conditions, become systemic and cause arthritis [3]. The disease causes economic losses by retarding growth and downgrading at slaughter [3]. Strategies to control this pathogen rely mainly in better management practices, improvement in housing conditions and antibiotic usage, whereas an effective vaccine is still not available [4].

Secreted proteins of pathogenic bacteria are key factors in host colonization. The analysis of these proteins, called secretome, can therefore permit the identification of new putative virulence factors that are fundamental for host invasion and survival in the environment within the host [5]. In this context, two-dimensional electrophoresis (2DE) along with peptide fingerprinting by mass spectrometry (MS) and subsequent protein identification have become a powerful method to unravel pathogenicity factors in microorganisms [6, 7].

We have recently reported a proteomic analysis of *M. synoviae* cell extracts in conventional Frey medium [8]. In the present work, we have grown *M. synoviae* in the same typical culture medium and then incubated the cells in a protein-free modified Frey medium as a strategy to indicate proteins that can be secreted to the medium by the bacteria.

## 2. Methods

### 2.1. *Mycoplasma synoviae* Cultures

*M. synoviae* strain 53 isolated from a broiler breeder was grown in the Laboratory of Genetics and Animal Health from EMBRAPA Swine and Poultry (Concórdia, C, Brazil) as described by Frey and coworkers (1968). The cells were cultured in Frey broth [9] supplemented with 12% swine serum, 0.1 g/L nicotinamide adenine dinucleotide (NAD), 0.1 g/L cysteine hydrochloride hydrate, 106 IU penicillin G, and 0.25 g/L thallium acetate at 37°C until the culture reached mid-log phase as indicated by color change and turbidity. Cells were pelleted by centrifugation and washed three times with “protein-free” modified Frey broth (3 g/L glucose, 0.1 g/L NAD, 0.1 g/L cysteine hydrochloride hydrate, 106 IU penicillin G, and 0.25 g/L thallium acetate) to reduce the level of contaminant proteins present in the growth medium.

Cells were cultured in this protein-free medium for 48 h and then centrifuged. Cell pellets were stored at −80°C and supernatants at −20°C. In order to assess the presence of contaminant proteins, equal volumes of protein-free Frey broth not exposed to *M. synoviae* cells were concentrated and analysed by two-dimensional gel electrophoresis (2DE) as described below.

### 2.2. Medium Concentration and Protein Extraction

Samples of 500 mL of protein-free Frey broth inoculated with *M. synoviae* were concentrated to 50 ml in a Quix Stand Benchtop concentrator through a GE Healthcare Xampler UFP-10-C-4X2MA membrane, 10,000 NMWC cutoff. Trichloroacetic acid (TCA) was added to a final concentration of 12% and samples were allowed to precipitate for at least 1 h on ice. Proteins were then pelleted by centrifugation at 16,000 x g for 20 min at 4°C and pellets were washed three times with cold acetone (−20°C). Samples were then airdried and solubilized in rehydration solution containing 7 M urea, 2 M thiourea, 4% CHAPS, 0.5% IPG buffer, pH 3–10, 18 mM dithiothreitol (DTT), and 0.002% bromophenol blue. Total protein concentration was determined using the 2-D Quant Kit (GE Healthcare) according to the manufacturer's instructions.

### 2.3. Two-Dimensional Electrophoresis

Samples in rehydration solution (250 *μ*g of total proteins) were applied on 13 cm long GE Immobiline DryStrip Gels (Ge Healthcare, Uppsala, Sweden), pH 3–10, and kept overnight at room temperature prior to isoelectric focusing (IEF). IEF was performed using the Ettan IPGphor 3 system (Ge Healthcare, Uppsala, Sweden) with maximum temperature set to 20°C with a total voltage of 17 kVh (500 V for 1 h, 500 V to 1000 V gradient in 1 h, 1000 V to 8000 V gradient in 2 hours 30 min, 8000 V for 30 min) and maximum current set at 25 *μ*A/strip. After IEF, strips were equilibrated for 20 min in 6 M urea, 30% glycerol, 2% SDS, 50 mM Tris-HCl, pH 8.8 containing 1% DTT, and another 20 min in the same solution containing 4% iodoacetamide instead of DTT. Equilibration treatments were performed under gentle shaking at room temperature. Second dimension was run in homogeneous 12% acrylamide gels. The gels were then fixed in 8% phosphoric acid and 40% ethanol and stained with Colloidal Coomassie Brilliant Blue G-250 (Bio-Rad, Hercules, USfA). The stained gels were scanned in a GE Healthcare ImageScanner III and images were analysed with the GE Healthcare ImageMaster 2D Platinum 7.2 software, having their molecular mass and isoelectric point calculated. Spots were excised manually and kept in 0.5% acetic acid.

### 2.4. In-Gel Digestion, Mass Spectrometry Analysis, and Protein Identification

Gel plugs were washed three times in 25 mM ammonium bicarbonate in 50% acetonitrile for destaining and dehydrated in 100% acetonitrile. Dry plugs were rehydrated with trypsin solution (10 *μ*g/mL Promega Trypsin Gold Mass Spectrometry Grade in 25 mM ammonium bicarbonate) and incubated for 12 to 16 h at 37°C. Peptides were extracted from gel plugs by three successive washes with 50% acetonitrile, 5% trifluoroacetic acid (TFA) for 30 min per wash and combined peptide extracts were dried in a vacuum concentrator (Eppendorf Vacuum Concentrator Plus). Peptides were resuspended in 0.1% TFA and mixed with equal volume of matrix solution (10 mg/mL *α*-cyano-4-hydroxycinnamic acid in 0,1% TFA in 1 : 1 acetonitrile/methanol), applied on the MALDI target plate, and allowed to crystallize at room temperature. Mass spectra were obtained in a mass range of 400 to 4,300 Da using a laser (337 nm, 200 Hz) as the ionization source. Each spectrum was formed by accumulated data from at least 900 shots, using the Bruker Daltonics flexControl 3.3 program. The instrument (Autoflex III MALDI-TOF Bruker Daltonics) was used in reflectron positive mode with an acceleration voltage of 19 kV. As external calibrant a Bruker Daltonics mix containing angiotensin II, angiotensin I, substance P, bombesin, ACTH Chip 1-17, ACTH Chip 18-39, and somatostatin 28, was used. Matrix and autolytic peaks of trypsin were used as internal calibration standards. Peptide lists were compared to the NCBInr database, using MASCOT search engine (http://www.matrixscience.com/). Search parameters were set as follows: enzyme: trypsin; taxon: Mycoplasma; fixed modifications: cysteine carbamidomethylation; variable modifications: methionine oxidation; mass tolerance: 100 ppm; monoisotopic spectra; 1+ charged masses, decoy enabled. MASCOT score was the main confidence factor for protein identification being also considered data of theoretical and obtained molecular weight and isoelectric point values, number of peptide matches, and the percentage of the total translated ORF sequence coverage by the peptides.

## 3. Results and Discussion

After 48 h of incubation in a protein-free Frey modified broth, protein extraction from cell pellets yielded the identification of 27 protein species, 8 of which were not detected previously from cell extracts of *Mycoplasma synoviae* cultivated in conventional Frey broth conditions [8]. These proteins are 6-phosphofructokinase, endopeptidase O, glucose-6-phosphate isomerase, leucyl aminopeptidase, phosphoenolpyruvate-protein phosphatase, serine/threonine protein kinase, transcription elongation factor GreA, and XAA-Pro aminopeptidase.

**Figure 1:**
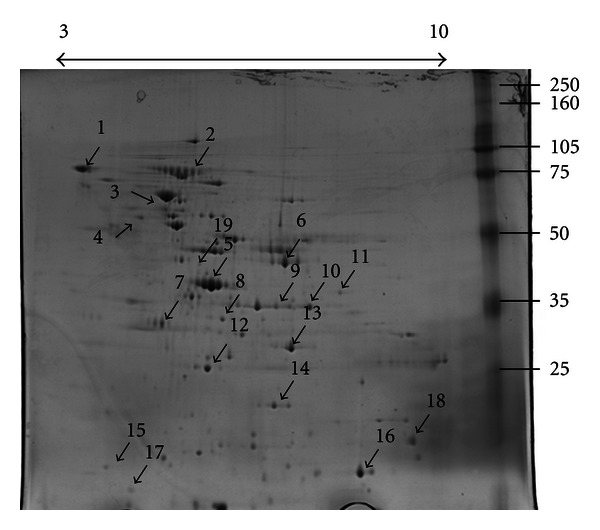
Two-dimensional gel electrophoresis profile of extracellular fraction of culture media incubated with *Mycoplasma synoviae*. Numbered arrows correspond to protein identities listed in Table 2.

In the extracellular fraction we have identified 3 uncharacterized proteins and 16 different characterized proteins (Figure 1 and Table 1), eight of which were not present in the cell fraction (Table 2), where their presence may have been masked by the presence of more prominent protein species. These proteins are cell division protein, DNA polymerase III beta subunit, elongation factor G, fructose-bisphosphate aldolase, and hypothetical proteins MS53_0566, MS53_0115, and MS53_0598. 

Acetate kinase and acyl carrier protein phosphodiesterase cause immunogenic responses in cattle infected by *M. mycoides* and *M. bovis*, respectively [10, 11]. As these proteins are able to promote an immune response in their hosts, it seems that they may be secreted or surface exposed during the infectious process. 

Thioredoxin reductase was described as surface exposed in *Trichomonas vaginalis*, and the authors suggest that they may change host mucus viscosity by modifying disulphide bonds [12]. 

Fructose-bisphosphate aldolase, which is surface exposed in *Enterococcus faecalis* [13], is immunogenic to cattle infected with *M. mycoides* [10]. 

Both subunits alpha and beta of pyruvate dehydrogenase component E1 from bacilli and mycoplasmas show immunogenicity [10, 14, 15], and the subunit alpha of this complex was shown to be surface-exposed in *M. genitalium* [14]. Moreover, both subunits alpha and beta have increased expression in *M. pulmonis* clones resistant to gentamicin and melittin [16]. 

Elongation factor G, which is secreted by *Paenibacillus larvae* [17] is produced by *Bacillus anthracis* and *B. thuringiensis* and are immunogenic to their hosts [15], as it happens in cattle infected by *M. bovis* [11] and *M. mycoides* [18] and chickens infected by *M. synoviae* [18]. 

EF-Tu has expression upregulated in the highly adhesive strain *Lactobacillus plantarum* WHE92 [19] and was demonstrated as extracellular in *Mycobacterium tuberculosis*, with capacity to bind to human plasminogen [20]. It is also secreted by *B. anthracis*, *B. cereus,* and *B. thuringiensis* with immunogenic effects [15, 21]. About 17% of total *M. pneumoniae* EF-Tu is associated with the membrane [22], being also demonstrated on the surface of *M. genitalium* and *M. pneumoniae* [14, 23]. It is immunogenic to mice infected with *M. genitalium* [14], to cattle infected with *M. mycoides* [10], to pigs infected with *M. hyopneumoniae* [24], and to chickens infected with *M. synoviae* [18]. 

As it happens to EF-Tu, the molecular chaperone DnaK shows upregulation in the highly adhesive strain of *L*. *plantarum* WHE 92 [19]. It was also observed on the surface of *E. faecalis*, along with fructose-bisphosphate aldolase [13], and in *M. tuberculosis *it has been demonstrated as extracellular, immunogenic, and capable to bind to human plasminogen [20, 25, 26]. *B. anthracis* DnaK is secreted, immunogenic and seems to act as virulence factor [21]. These evidences suggest that the chaperone DnaK is secreted by a number of pathogens and may be important in the disease process by mediating adhesion to host tissues. Pigs infected with *M*. *hyopneumoniae* [24], cattle with *M. mycoides* [10] and *M. bovis* [11], mice with *M. genitalium* [14], and chickens infected with *M. synoviae* [18] all rise antibodies against the molecular chaperone DnaK. 

Finally, enolase is an enzyme widely described as secreted or surface exposed in several microorganisms of the genera *Paenibacillus*, *Bacillus*, *Lactobacillus,* and *Streptococcus* [17, 27–30], showing immunogenic properties [15, 21, 29] and ability to bind to fibronectin [30, 31]. Enolase is also shown to be a virulence factor in *P*. *larvae* [32] and is found on the surface of *L*. *crispatus* in a pH-dependent way, being released to the medium at pH close to its isoelectric point or more alkaline [28]. In Mycoplasmas it has been detected on the surface of *M. gallisepticum* and *M. fermentans*, in both cases able to bind to plasminogen [33, 34]. In *M. suis* its role in cell adhesion was clearly demonstrated by inserting the gene in *Escherichia coli* that once transformed became able to bind to swine red blood cells [35]. 

## 4. Concluding Remarks

We conclude that by incubating *M. synoviae *cells in protein-free modified Frey medium we were able to produce clearly different gel profiles using protein extracts from the cellular and extracellular fractions, showing exclusive proteins for each fraction. In the extracellular fraction we have found proteins that are originally described as cytosolic but in more recent studies are shown in other pathogenic microorganisms to be either surface exposed or secreted, immunogenic or able to bind host components as fibronectin or plasminogen, thus participating in the infectious process. These proteins are acetate kinase, elongation factor G, elongation factor Tu, acyl carrier protein phosphodiesterase, fructose bisphosphate aldolase, thioredoxin reductase, DnaK, both alpha and beta units of pyruvate dehydrogenase E1 component, and enolase. These evidences suggest that they may be secreted by *M. synoviae* and can thus be implicated in disease process.

## Figures and Tables

**Table 1:** Cellular proteins identified in *Mycoplasma synoviae*.

Identity	gi entry	MASCOT	Observed		Theoretical	
		RowSpanEmpty	Score	MW	pI	MW	pI
6-phosphofructokinase	gi∣144575058	96	33747	9.24	33891	8.67
Acetate kinase	gi∣144575215	120	37945	7.61	44515	7.10
Acyl carrier protein phosphodiesterase	gi∣71894112	96	13718	8.42	22687	7.74
Cell division protein	gi∣71894355	106	70988	3.51	62539	4.38
Elongation factor EF-Ts	gi∣71894429	91	31657	5.87	31938	5.72
Elongation factor Tu	gi∣71894677	126	39239	5.50	43230	5.61
Endopeptidase O	gi∣71894512	162	64809	7.21	42295	6.08
F0F1 ATP synthase subunit beta	gi∣71894420	89	47222	6.01	50804	5.76
Glucose-6-phosphate isomerase	gi∣71894495	65	44765	7.56	48990	6.99
Hypothetical protein MS53_0316	gi∣71894332	68	73092	4.86	81668	5.48
Leucyl aminopeptidase	gi∣71894176	151	47812	5.65	52277	5.83
Molecular chaperone DnaK	gi∣71894366	115	54392	5.37	65096	5.17
Phosphoenolpyruvate-protein phosphatase	gi∣71894534	103	58361	5.23	63753	5.19
Phosphopyruvate hydratase	gi∣71894034	108	45607	7.20	63753	5.19
Phosphotransacetylase	gi∣71894663	98	34133	6.46	35000	6.06
Putative lipoprotein	gi∣144574996	124	92020	4.96	100677	5.13
Putative lipoprotein	gi∣71894364	103	96688	5.71	112804	5.94
Putative lipoprotein	gi∣71894528	66	81447	5.73	85199	5.72
Putative trigger factor	gi∣71894613	126	55239	5.26	53932	5.31
Pyruvate dehydrogenase E1 component, beta subunit	gi∣144575045	126	30000	7.41	35853	6.73
Ribonucleotide-diphosphate reductase subunit beta	gi∣71894414	153	34317	6.10	39239	5.09
Serine/threonine protein kinase	gi∣47459394	77	37945	6.15	37008	9.35
Single stranded binding protein	gi∣71894544	155	15419	4.29	21239	4.50
Thiol peroxidase	gi∣71894383	125	8897	5.45	18263	5.93
Thioredoxin reductase	gi∣71894606	107	32559	6.32	27439	5.37
Transcription elongation factor GreA	gi∣71894394	66	8597	4.69	17916	4.95
XAA-Pro aminopeptidase	gi∣71894172	78	37710	6.73	40194	6.12

**Table 2:** Proteins present in the extracellular fraction of culture media incubated with *Mycoplasma synoviae*.

	Identity	gi entry	MASCOT	Observed		Theoretical	
			RowSpanEmpty	Score	MW	pI	MW	pI
(1)	Cell division protein	gi∣144575078	97	77515	3.66	62539	4.38
(2)	Elongation factor G	gi∣71894071	79	72218	5.25	77259	5.28
(3)	Molecular chaperone DnaK	gi∣71894366	61	63596	3.51	65096	5.17
(4)	Cell division protein	gi∣71894355	120	79326	3.49	62539	4.38
(5)	Elongation factor Tu	gi∣71894677	111	37523	5.43	43230	5.61
(6)	Phosphopyruvate hydratase	gi∣71894034	62	43611	6.01	49330	6.59
(7)	Ribonucleotide-diphosphate reductase subunit beta	gi∣71894414	127	34255	3.88	39239	5.09
(8)	Phosphotransacetylase	gi∣71894663	169	34605	4.94	35000	6.06
(9)	Pyruvate dehydrogenase E1 component, alpha subunit	gi∣71894294	68	36239	5.87	42131	6.20
(10)	Acetate kinase	gi∣144575215	80	36194	6.10	44515	7.10
(11)	DNA polymerase III beta subunit	gi∣71894027	104	38563	6.96	43399	7.62
(12)	Thioredoxin reductase	gi∣144575189	110	31039	4.67	27439	5.37
(13)	Pyruvate dehydrogenase E1 component, beta subunit	gi∣144575045	224	32423	6.12	35853	6.73
(14)	Fructose-bisphosphate aldolase	gi∣71894369	76	26539	5.82	31237	6.27
(15)	Single stranded binding protein	gi∣71894544	88	14149	3.00	21239	4.50
(16)	Acyl carrier protein phosphodiesterase	gi∣71894112	94	13164	7.29	22687	7.74
(17)	Hypothetical protein MS53_0566	gi∣144575177	90	10711	3.36	20869	4.96
(18)	Hypothetical protein MS 53_0115	gi∣71894139	103	18926	8.23	21059	8.77
(19)	Hypothetical protein MS 53_0598	gi∣71894608	97	42120	4.67	44307	5.60
